# A framework for developing generic implant safety procedures for scanning patients with medical implants and devices in MRI

**DOI:** 10.1093/bjr/tqae232

**Published:** 2024-11-13

**Authors:** Jonathan P Ashmore, Sarah J Prescott, John McLean, Daniel J Wilson, Geoff Charles-Edwards, Peter Wright, David Grainger, Gareth J Barker, Alexandra J Lipton, Rachel Watt, Deepa Gopalan, Mark R Radon

**Affiliations:** Department of Medical Physics and Bioengineering, NHS Highland, Inverness, IV2 3UJ, United Kingdom; Radiology Physics Department, University Hospitals of North Midlands NHS Trust, ST4 6QG, United Kingdom; Representative for Institute of Physics and Engineering in Medicine, York, YO24 1ES, United Kingdom; MRI Physics, Department of Clinical Physics and Bioengineering, NHS Greater Glasgow and Clyde, Langlands Dr, Glasgow, G51 4LB, United Kingdom; University of Glasgow, Glasgow, G51 4LB, United Kingdom; Representative of the NHS Scotland MRI Physics Group, Glasgow, G51 4TF, United Kingdom; Medical Physics and Engineering, Leeds Teaching Hospitals NHS Trust, Leeds, LS9 7TF, United Kingdom; Guy's & St Thomas' NHS Foundation Trust, London, SE1 7EH, United Kingdom; King’s College London, London, WC2R 2LS, United Kingdom; Royal Marsden NHS Foundation Trust, London, SM2 5PT, United Kingdom; Institute of Cancer Research, London, SM2 5NG, United Kingdom; University Hospitals Plymouth NHS Trust, Plymouth, PL6 8DH, United Kingdom; Medicines and Healthcare Products Regulatory Agency, London, E14 4PU, United Kingdom; Department of Neuroimaging, Institute of Psychiatry, Psychology & Neuroscience, King’s College London, London, SE5 8AB, United Kingdom; Representative for the British and Irish Chapter of the International Society for Magnetic Resonance in Medicine, Concord, California, 94520, United States; Society of Radiographers, London, SE1 2EW, United Kingdom; Circle Health Group, Albyn Hospital, Aberdeen, AB10 1RW, United Kingdom; Representative for The British Association of MR Radiographers; Imperial College Healthcare NHS Trust, London, W110HS, United Kingdom; Representative for The Royal College of Radiologists, London, WC2A 3JW, United Kingdom; The Walton Centre NHS Foundation Trust, Liverpool, L9 7LJ, United Kingdom; Representative of The British institute of Radiology, London, EC1N 6SN, United Kingdom

**Keywords:** MRI, Implant, Safety

## Abstract

UK guidelines for MR safety recommend that MRI departments refer to the implant manufacturer for advice regarding the MRI safety of scanning patients with an implantable medical device prior to scanning. This process of assuring safety can be time consuming, leading to delays and potential cancellations of a patient’s MRI. Furthermore, at times the implant cannot be identified, or the implant manufacturers cannot provide up to date MRI safety information. The purpose of generic implant safety procedures is to define a process for managing patients with certain types of implants where the risk from scanning is low. This process incorporates scope for an evidence-based risk-benefit decision to scan some groups of patients under locally approved conditions, without seeking to identify the exact make and model of the implant and subsequent assurance of MR safety from the implant manufacturer. This publication provides best practice recommendations from a multi-professional working group for the development of these procedures. It is supported by The Institute of Physics and Engineering in Medicine, The Society of Radiographers, The Royal College of Radiologists, The British Institute of Radiology, The British Association of MR Radiographers, The International Society of Magnetic Resonance in Medicine British and Irish Chapter, and the NHS Scotland MRI Physics Group.

Key messages

- The working group advocates MRI scanning of certain implants without manufacturers’ assurance of safety (so-called generic or blanket scanning).
- Such scanning should be undertaken through a generic implant safety procedure (GISP).
- This procedure should incorporate an Evidence Review, Risk Assessment, Procedure Statement/Workflow and formal approval within the relevant institution’s governance framework.
- The procedure should undergo formal audit and review on a periodic basis.
- This article is aimed at radiographers, clinical scientists, and other imaging professionals who develop MRI implant procedures. It is designed to be a resource for institutions to refer to when creating GISPs.
- A GISP can support MRI departments to scan low-risk patients under pre-defined, institutionally approved, procedures, should they wish to do so.

## Introduction

While MRI is in general a very safe technology a number of serious incidents and safety alerts continue to highlight the importance of considering implant safety prior to scanning.^1–6^

The UK Medicines and Healthcare products Regulatory Authority (MHRA) Safety guidelines for MRI Equipment in Clinical Use recommend that MRI departments should develop a procedure for the identification, documentation, imaging, and provision of any necessary aftercare for patients with implantable medical devices undergoing an MRI examination.^7^ It also states that users should refer to the implant manufacturers for advice on the safety of each implantable medical device. The UK quality standards in imaging provides similar guidance, highlighting that “There must be a system of work in place for the management of implanted devices”.^8^ The American College of Radiology (ACR) Manual on MRI Safety,^9^ states that MR safety infomation related to a device should not be assumed when it is not clearly documented in writing. Other National Guidance Documents have similar recommendations.^10^^,^^11^

Managing patients according to these national guidelines can require significant resourcing as a large proportion (21%) of patients present to MRI with an implanted medical device.^12^ A similar audit undertaken at 2 of the authors’ institutions found that 25% of 97 consecutively screened patients had medical implants at the first institution and 29% of 152 consecutively screened patients had medical implants at the second institution.

Identifying the implant details and subsequently manufacturers’ MRI safety labelling can often prove difficult, leading to delays and potential cancellation of scans. However, for certain implant categories (particularly passive implants), there may be strong evidence that the risk associated with scanning patients who have implants in this category is very low. In such cases, it may be appropriate to define general workflows for how these patients are managed without needing to explicitly identify the implant make and model and confirm the MRI safety from the device manufacturers. Instead, all patients with implants from the category will follow the same generic scanning workflow. These workflows or “generic implant safety procedures” (GISPs), can apply to a particular implant category and/or patient group with strict inclusion and exclusion criteria. In practice, many institutions are likely already undertaking a similar process as described above, with non-medical implants such as cosmetic implants/body modifications, but potentially also with medical implants such as fixed internal orthopaedic implants.

In a recent survey of UK NHS Hospitals, it was found that 89% of 86 responding institutions were already using GISPs for at least one implant type.^13^ However, respondents highlighted that multiple barriers exist which hinder the development of GISPs including concerns over completeness of the procedure, difficulties obtaining evidence, lack of time to develop the procedure, and concerns over compliance with MHRA guidelines.

For the purpose of this article, we will only consider the governance framework and process of developing a GISP for medical implants although many of the steps outlined here could be applied to non-medical implants (eg, non-removable piercings).

Some institutions in the United Kingdom, which are already using GISPs, have reported their experience with setting these up.^12^^,^^14^ Outside the United Kingdom, others have proposed generic scanning of several implant categories including coronary stents, heart valves, and vascular access ports.^15–17^ A published expert consensus utilised a modified Delphi method to provide consensus recommendations for scanning of the 10 most frequently questioned devices.^18^ A Dutch guideline publication described a process to develop modules for implants very similar to the GISPs we propose here.^19^ There is currently guideline modules for heart valves, annuloplasty rings, or mitra clips and for aneurysm clips.^20^ Recently, a population-based analysis has been applied to develop recommendations for managing patients with implants in MRI research studies within Germany,^21^ where an expert committee was formed to review individual implant MRI suitability queries for research subjects. After 1 year, the expert committee considered the outcomes for all queries and generated a list of medical implants which could subsequently be considered as safe for MRI.

**Figure 1.**
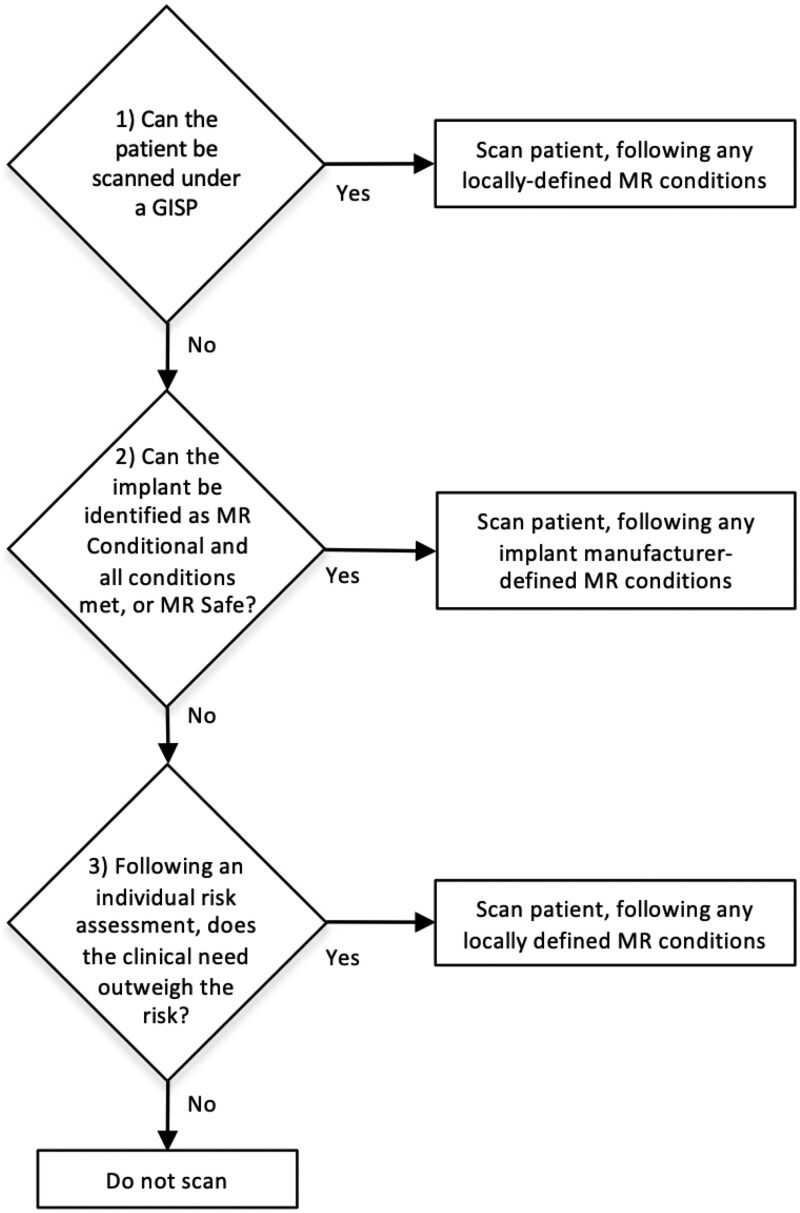
Overall workflow for clinical MRI patients highlighting how each implant should be managed.

Having an established workflow for managing patients with implants is important to ensure a streamlined, effective, and safe MRI service. Figure 1 highlights an example of one such workflow indicating how GISPs can fit into the overarching patient implant management structure within an MRI department.

### Benefits and risks of GISPs

The risks and benefits of implementing a GISP within an MRI department are highlighted in Table 1.

**Table 1.** Benefits and risks of implementing a GISP.

Benefits	Risks
Facilitates scanning when implant information is not available and reduces the need for multidisciplinary team review of the specific patient case or onward referral to a larger more experienced centre. Facilitates immediate scanning when implant information takes some time to obtain, removing delays to patient care. Immediate scanning avoids wasted scan slots leading to an improved and more cost-effective MRI utilisation. Reduces the need for staff resources to obtain and evaluate specific implant information. Allows greater emphasis to consider implants not covered by the GISP and ensures the safety focus is on these implants which are typically higher risk. Evidence based and therefore a more proportionate attitude towards risk. Supports MRI staff by providing clearly defined procedures. Facilitates a consistent approach to scanning implants within a department or imaging network. This in turn helps manage patient expectation and improves confidence in the healthcare system.	Unknowingly scanning an MR Unsafe implant, for example an implant previously unrecognized. Unknowingly scanning an implant where the MRI safety information has changed such that it is no longer safely scanned under a GISP. Knowingly scanning an MR Conditional device under a GISP outside its MRI conditions (also termed “off-label”) with the associated institutional liability. When following a GISP, implants not disclosed by the patient at screening might not be discovered, whereas identifying implant specifics in patient notes can highlight inaccuracies in the patients account of their own medical history. Confusion regarding exactly what implants or patient groups a GISP covers. When make and model are identified this ambiguity is removed (eg, an active orthopaedic implant mistakenly categorised as a passive orthopaedic implant).

Given the potential risks, a robust process needs to be in place for developing, implementing and reviewing a GISP. This should ensure the risks are understood and when appropriate, mitigation measures and controls are in place, to reduce the risks to an acceptable level.

Consent is already required for all patients attending MRI and appropriate procedures should be in place (see eg, QSI XR 502 consent quality standard^8^). Institutions should also consider if the patient should be explicitly informed of the risks associated with a GISP or the potential that a patient’s specific device may be scanned off-label.^22^ In cases where it is appropriate, we suggest the following statement:

“Up-to-date expert advice suggests that many of the items covered during the MRI safety screening are generally regarded as safe to scan, even if the original instructions for the implant did not state this. We will always discuss any concerns with you prior to your MRI but if at any point you have any questions, then please ask a radiographer for more information.”

A statement such as this could be provided on the patient screening form, the letter sent to the patient prior to their appointment, or verbally to the patient should they have an implant/device which is to be scanned under a GISP. The SOR provide further useful resources for radiology departments regarding consent.^23^

In the subsequent sections of this article, we offer consensus guidance to enable more effective management of patients with certain types of implants, allowing patients to be safely scanned without unnecessary delays and with reduced information gathering burden to the MRI department. We outline a robust evidence-based approach which could be considered best practice, but we acknowledge many institutions that already implement generic scanning procedures may well already be undertaking their own safe approach to scanning these patients.

## Framework for creating GISPs

### Framework overview and personnel

**Figure 2.**
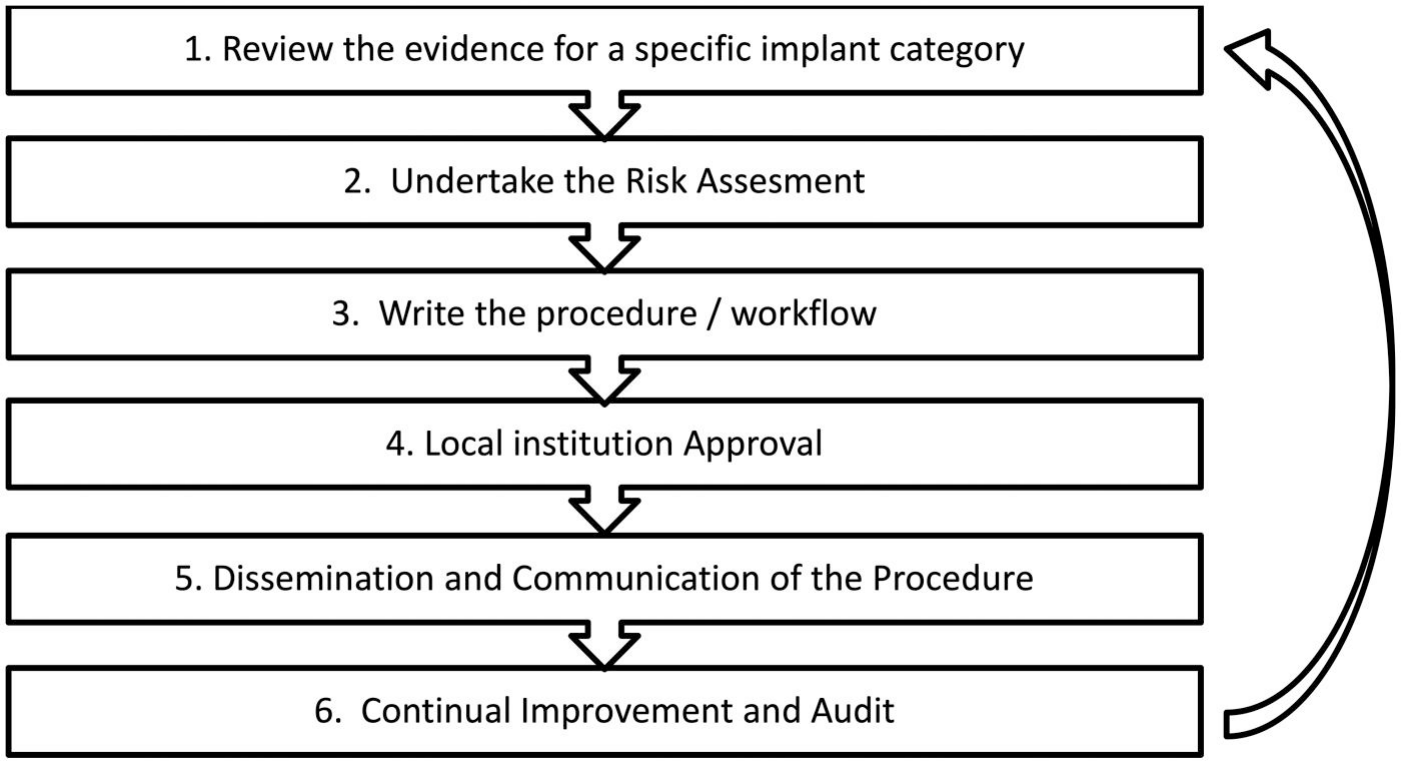
An example of a governance framework for creating GISPs is shown. Abbreviation: GISPs = Generic Implant Safety Procedures.

Figure 2 shows an example of a governance framework which could be used to establish a GISP, further details are provided in the framework steps discussed in the remainder of this article. The process should involve a number of personnel, each having specific expertise (see Table 2).

**Table 2.** Roles for personnel involved in creating and implementing GISPs.

Person	Key tasks and responsibilities
MR Safety Expert (MRSE)^a7^	Involved in researching and developing the various aspects of the procedure Provide scientific input to interpret the evidence Dissemination and training of MRI staff on the procedure Remain abreast of up-to-date MRI safety information between review cycles—allowing for immediate review should a relevant safety concern be publicised Advise on the imaging sequences/protocols required (eg, SAR and B1+rms considerations) Overseeing the framework for development and implementation of GISPs
MR Responsible Person^b7^	Involved in researching and developing the various aspects of the procedure Dissemination and training of MRI staff on the procedure Review the procedure to ensure it is practical and implementable within an MRI department Remain abreast of up-to-date MRI safety information between review cycles—allowing for immediate review should a relevant safety concern be publicised Development of imaging sequences/protocols required (eg, SAR and B1+rms considerations)
MRI Clinician (radiologist or imaging cardiologist^c^)	Involved in researching and developing the various aspects of the procedure Provide clinical input to interpret the evidence
Implanting surgeon, interventionalist, or device specialist	Provide specialist clinical expertise regarding specifics of an implant (eg, how it is used, where it is implanted etc.) Provide information regarding locally implanted devices
MRI radiographer	Review the procedure to ensure it is practical and implementable within an MRI department Safe use of the procedure for scanning patients with implants Development of imaging sequences/protocols required (eg, SAR and B1+rms considerations)
Implant manufacturer (eg, local sales rep)	Provide information regarding locally implanted devices Provide details regarding MRI safety status of implants and/or further details regarding design and composition of an implant

a Some tasks may be undertaken by persons other than the MR Safety Expert but whom have the required scientific technical and clinical knowledge (eg, clinical scientists).

b Typically superintendent radiographer.

c A consultant radiographer could also undertake this role.

### Framework step 1: Review the evidence for a specific implant category

A wide range of different evidence sources should be consulted when creating a GISP. Suggested sources are summarised in Table 3 together with a comment on the quality and pitfalls of using the evidence source. A more complete description of each evidence source is provided in the Supplementary Information. Also included in the Supplementary Information is a discussion on how to critically review the evidence to understand the larger context of how this would relate to the risk associated with a GISP and methods to mitigate this risk. When first writing a GISP it is strongly recommended users review this Supplementary Information. The level of detail required from each evidence source will vary depending on the category of implant. Some implants where the risk is expected to be low will require less evidence to be compiled for the GISP.

**Table 3.** Potential sources which can provide evidence to incorporate into the detailed review.

Source	Description of evidence	Quality of evidence and Pitfalls
Online MRI safety databases	Provides non-exhaustive list of devices with an indication of the MRI safety status.	Does not represent formal manufacturer labelling and at times lacks manufacturer conditions. Typically, does not contain MR Unlabelled implants.
Local implanting teams	Provides a list of locally implanted devices and potentially the associated instructions for use (IFU) with the MRI safety status. Also provides clinical information on implanting techniques.	Provides route to formal manufacturer labelling on devices implanted in patients from the local institution. Hence constitutes high-quality evidence for likely, locally scanned patients. However, at times the full suite of locally implanted devices is difficult to obtain.
Implant manufacturers	Provides list of current products, their MRI safety status and conditions for MR Conditional devices. Can provide general statements about MRI safety for a range of devices. Can help identify make and model of locally implanted devices and provide evidence of MRI safety testing carried out. Also provides clinical information on implanting techniques.	Robust source of formal labelling information. Historical implants, however, can have outdated labelling and at times overly restrictive labelling can be in place from the manufacturers. Hence, scientific scrutiny of test data can be helpful.
Peer-reviewed literature	Provides makes and models of devices, testing data for specific makes and models, consensus statements, guidelines, and expert opinions. Provides information regarding device composition, reports of adverse incidents and can be useful to provide information regarding the historical evolution of an implant category.	Robust peer reviewed evidence (particularly for consensus statements, guidelines etc.). Testing methodologies across the literature and reported outcomes can vary. Single case reports of incidents at times require scientific scrutiny. Consider the citing literature for further discussions of case incident reports.
Internet search (non-peer-reviewed literature)	Provides list of manufacturers of devices and information on new devices on the market. Provides potential reports on any adverse incidents. Can provide procedure information from other MRI centres (eg, hospital websites).	Non-peer reviewed evidence and procedures from other centres may be provided without background evidence and/or may be outdated.
Regulatory Medical Device and Clinical Trials Databases	Provides information regarding MRI safety status of a specific medical devices for sale on the market and used in clinical trials.	Databases are robust and broad reaching as manufacturers are required to submit details of all new devices on the market. Historical devices (circa > 10 years old) not included.
Regulatory Professional and Standards bodies	MHRA provide field safety notices (changes in MRI safety status and conditions). FDA MAUDE database provides adverse incidents. Various bodies provide national guidelines for MRI safety which includes implant information. Standards bodies provide MRI testing criteria.	Robust source of information for labelling changes to devices and for national guidelines on the management of devices. Incident reports are typically anecdotal, not peer reviewed and at times are not submitted by health care professionals.
Local MRI safety databases	Provides further information regarding devices and historic conditions related to patients who have been referred for a local MRI scan.	Are often historical and do not contain the most up to date implants.
Other sources of information	Email groups, social media platforms with searchable archives provide anecdotal information. Policies and experiences can also be shared by personal communications between professionals.	Requires a high level of caution as information from these sources is anecdotal and will not have undergone robust scientific scrutiny.

Sources are listed in the order which, from experience the authors feel the evidence review would be undertaken, defined through the combination of the quality of evidence and ease of access. The list order does not represent level of authority.

### Framework step 2: Undertake the risk assessment

Once the evidence has been reviewed, each of the potential risks can be assessed separately. Many of these will be generic across all GISPS (see risks discussed in the section Benefits and risks of GISPs) but in some cases, risks specific to an implant may be identified. For instance, in the case where a patient presents suggesting they have a non-programmable shunt, there is a risk that their device has been mis-identified and is in fact programmable. To mitigate these risks, asking additional standardised questions can provide further assurance that a patient has the type of implant covered in the GISP. Furthermore, when implementing a GISP, given that the patient’s record is no longer investigated the risk of missing additional procedures or implants which were in the medical records but not disclosed by the patient is increased. The person undertaking the screening must feel confident of the accuracy of the patient’s own medical history, and if any doubt persists medical records should still be investigated. In instances where an implant has overly conservative MRI conditions, the off-label risk should be evaluated (further information is provided in the Supplementary Information “Guidance on Evidence Sources”). In some cases, it can be prudent to have a specified time-frame built into the GISP (eg, when there was a known period during which an MR Unsafe implant was on the market). The Dutch guidelines which advocate the scanning of aneurysm clips implanted within the Netherlands on or after the year 2000 are a good example of this.^19^ It also may be the case that there is less confidence in knowing the MRI Safety details of devices implanted prior to a particular date.

Even if the evidence review finds no safety concerns, there remains a risk because any research undertaken is unlikely to be completely exhaustive. However, for implant categories with no known reported incidents, this can be a compelling empirical dataset in its own right, particularly for those categories which are already scanned generically by numerous institutions. The survey results provided by McLean et al^13^ highlight a number of such implant categories generically scanned at institutions across the United Kingdom. This anecdotal evidence, combined with all other evidence sources, can give an indication of the overarching risk, which for a GISP is typically extremely low. The consequence of an incident should a GISP be implemented should also be considered. This can vary between implants, and a discussion with the full multidisciplinary team is appropriate to understand the possible outcomes. It should be noted as the frequency of adverse events in MRI is very low, the threshold for what is considered an acceptable risk may be lower than in other areas of medicine.

The risk of implementing the GISP in different MRI settings should also be considered, for example should the GISP restrict scanning to normal mode and/or 1.5 T only? It might seem prudent to limit the scanning to the most restrictive conditions possible, but this might have clinical implications (eg, reduced diagnostic efficacy when not scanning at 3 T). Hence the risk assessment should consider the implications of scanning the device under various conditions which can then inform exactly which restriction should be in place for the Procedure Statement/Workflow.

### Framework step 3: Writing the Procedure Statement/Workflow

Once all the evidence has been reviewed for a particular implant type, a procedure statement or workflow can be established. Any restrictions that need to be put in place should be considered, such as geographical or time period restrictions and practical aspects such as limiting SAR or field strength (eg, 1.5 T only). For institutions with multiple scanners operating under a single local governance, it can be worthwhile highlighting on which individual scanners the GISP can and cannot be applied. This may need to be revised upon scanner replacements or upgrades. The Procedure Statement/Workflow should address possible confusion between implant types (eg, programmable vs non-programmable shunts), potentially via additional standardised questions to ask the patient when screening, or through review of previous imaging. Any exclusions should be clearly highlighted, for example if the procedure relates to aneurysm coils then it may highlight that aneurysm clips are not covered by the procedure.

An example procedure developed by the Scottish Clinical Engineering and Medical Physics Network and the implementation within one of the networks institutions can be found here www.mriphysics.scot.nhs.uk/implant-safety-policies/intrauterine-contraceptive-devices. The template used to develop this GISP which follows the framework described in this article can be found in the Supplementary Information.

### Framework step 4: Local institution governance approval

As a minimum the procedure should be reviewed and agreed by the local MR Safety Expert, MR Responsible Person, lead clinician for MRI safety, and lead MRI radiographer (who may not all be different people). A suitable forum for this to be approved would be at the institution’s MRI Safety Committee. Given GISPs may involve scanning devices off-label, institutions should consider getting high level approval (eg, radiation or radiology governance group) for the GISP in principle and a lower level (eg, MRI safety committee) approval for individual GISPs. It is important, however, that the procedure including the risk assessment is documented and formally signed off in accordance with the relevant institution’s local governance procedures, the institution being the responsible legal entity. Departmental management should be made aware which may help facilitate implementation, ensure resourcing requirements are available and that there is compliance with other institutional policies.

### Framework step 5: Dissemination, communication, and implementation of the procedure

It is essential that the procedure is effectively communicated and made available to staff. Given it may differ from the implant scanning practices currently in place, it is important to educate MRI radiographers and other relevant staff on any new processes that the GISP may involve. A GISP should be implemented by an appropriately skilled healthcare professional who fully understands the procedure and is experienced in identifying cases where patients might be giving an inaccurate account of their own medical history. This training is particularly important for locum radiographers whom, when starting, will likely be unaware of the local procedures. As such, an induction and sign off process should be undertaken for all new radiographers educating them on which GISPs are in place.

Given the traditionally established approach is to identify make and model of implant, it is understandable that radiographers may have concerns in allowing patients to be scanned without this full assurance. Education can hopefully mitigate any concerns, as can knowledge that the procedure has been approved within the institution’s governance framework. The governance forums and relevant management can assure concerned staff that GISPs have been developed appropriately, and there are suitable resources in place for the ongoing development and safe implementation. As discussed previously, consent is required where there is potential to scan devices off-label and an example statement is provided in the section Benefits and risks of GISPs. Staff may need support and training on how best to inform patients of the risks according to their local policy and documentation and screening forms may need to be updated accordingly.

To ensure transparency, all aspects of the GISP (eg, the documented evidence and risks assessment etc.) should be available to any staff member within the institution who requests it.

There is often interest in sharing GISPs between institutions helping to avoid the need to repeat the research required. Indeed, several examples exist where procedures are publicly available from healthcare institutions (eg, www.mriphysics.scot.nhs.uk/implant-safety-policies), professional insitutions,^19^ and other groups.^15–18^ It should be noted however that direct implementation of another institution’s procedure comes with risk, particularly when the full evidence associated with the procedure is not provided. Any such implementation should always be carefully considered and undertaken through an appropriate governance process. Future work for this multi-professional group is to create publicly available GISPs, which follow the framework reported in this manuscript, provided as a resource for healthcare institutions.^24^

### Framework step 6: Continual improvement and audit

Any procedure(s) implemented should be reviewed periodically, with a review period dependent on the level of risk. They should be reviewed immediately if new and significant information comes to light (eg, a new implant is marketed with significant MRI safety concerns) or if there is evidence of an adverse event or near miss. It should be the responsibility of at least one person who is involved with creating and reviewing the GISP to remain abreast of the latest MRI safety information as part of their continued professional development (eg, by attending external MRI safety meetings and conferences). Device safety updates relating to MRI are disseminated by the MHRA (www.gov.uk/drug-device-alerts) and the FDA (https://www.fda.gov/medical-devices/medical-device-safety). These are often further circulated to the community via online forums such as the UK MRI safety jiscmail group or the MRI safety facebook group. The UK Health Security Agency (UKHSA) has developed a national error reporting system which provides a taxonomy for incident reporting in radiology.^25^^,^^26^ This includes a category for reporting incidents resulting from inadequate procedures in MRI. When this system is live we recommend incidents associated with GISPs be recorded to provide a national archive which may help highlight any potential risks associated with specific GISPs.

To help stay informed of newly implanted devices, it can be helpful to develop good relationships with implanting departments from your institution and with the device manufacturer representative.

The procedure review should include (1) checking the scientific literature for any new evidence, (2) rechecking online databases for new or changed safety status of implants, and (3) identifying any changes to locally implanted devices if these were part of the original evidence.

Many of the risks identified in the section Benefits and risks of GISPs can be mitigated by ensuring the process embeds a culture of audit and procedure improvement (eg, see the Quality Standards for Imaging^8^). All staff involved in developing and utilising the procedures should have an ongoing role in procedure improvement, for example, they may become aware of new devices or changes to device safety conditions. Crucially, they should report any adverse events immediately to those involved in reviewing the procedures, and subsequently to the regulatory authority.

## The use of GISPs in research

Given the high percentage of the general population with implants, it is inevitable that such persons will be recruited as participants in research studies involving MRI. While for clinical patients there is an implicit benefit to performing MRI scans, for research and trial participants, it is less likely there is a direct benefit. However, as the risks of GISPs are typically low, we recommend that institutions performing research MRI consider adopting GISPs in a similar manner to clinical MRI units.

In a research setting, research *ethics* and research *governance* must be considered. Research participants give informed consent to all parts of the research process, and in general should be aware of any risks involved and the procedures that have been put in place to minimise the risks.

In some cases, because of the inclusion criteria of the study, it may be likely that participants have a particular category of implant. Information on the procedure for managing such participants and any associated risk could be included in the ethical approval process and the participant information sheet (PIS). A statement like that provided in the section Benefits and risks of GISPs may be appropriate to use in this setting. Consent could then be sought in the usual way as part of the overall consent procedure for the study. Where it is unlikely to be known that participants would have a particular category of implant (ie, the implant is unrelated to the inclusion criteria), it would be difficult to provide details of how all possible implants would be managed for the ethical approval process and in the PIS. Researchers may wish to state in the ethical approval documentation that participants with implants would be scanned under the department GISPs as per clinical patients. Alternatively, if the risks to the study participants from the use of GISPs is appropriately low (eg, in comparison to risks encountered in everyday life) then the consent process from the standard MRI safety screening alone (which for example includes the statement provided in the section Benefits and risks of GISPs) may be adequate. For research taking place in an NHS setting, Health Research Authority (HRA) review may also be necessary.

It is worth noting that GISPs developed for clinical use have potential to invalidate the results of a research study due to restrictive conditions such as limiting the scan to normal mode. This may lead to undesirable variation in the results between participants with and without implants. In such cases GISPs could be developed specifically for the research institution allowing the same scan conditions to be applied to participants with and without implants. The risk assessment (as per Framework step 2: Undertake the risk assessment) should provide a level or risk which can be reviewed by the research team and included in the ethical review and PIS if required. This idea of developing a GISP to suit the setting is similarly highlighted in Framework step 2: Undertake the risk assessment where limiting GISPs to normal mode vs first level is discussed.

Dissemination and communication of the procedure (as per Framework step 5: Dissemination, communication, and implementation of the procedure) is of particular importance in research studies to ensure staff can address any questions raised. A research participant has the right to withdraw from a study (or any element within it) and having been informed of the risks of undergoing an MRI, participants with an implant may be more likely to withdraw.

## Conclusion

The apparent prevalence of GISPs within the UK MRI community suggests that scanning patients with certain categories of implants generically is well established and will most likely not be reversed. Hence, we advise that scanning patients with implants using a GISP is done so within a suitable governance framework. This should include appropriately documented evidence, risk assessments, as well as review, approval, and subsequent audit. Developing a GISP is a multidisciplinary process requiring input from varied personnel. A GISP will always have residual risk and this should be weighed against the potential benefit, not only to the patient but also to the institution that must provide the staff resourcing if implants are to be managed without a GISP in place. It may also be appropriate to apply GISPs to research participants, if doing so, this should be done within the appropriate research framework. This guidance has been developed for the UK context, however we are aware that GISPs (or their equivalent) are implemented within other international settings, and as such a similar framework as we describe here may be appropriate. Governance requirements in other countries however may differ and therefore the local regulatory and professional context should be considered.

## Supplementary Material

tqae232_Supplementary_Data
