# Supplementary material for: A framework for developing generic implant safety procedures for scanning patients with medical implants and devices in MRI
Source: Br J Radiol. 2024 Nov 13;98(1167):336–44. doi: 10.1093/bjr/tqae232 (PMC11840167; doi:10.1093/bjr/tqae232)
Supplement: tqae232_Supplementary_Data [file tqae232_supplementary_data.docx]

# Example GISP Template

| Document Details | |
| --- | --- |
| Author |  |
| MRSE |  |
| Radiographer |  |
| Other (add rows as required) |  |
| Authorisation date (MR safety committee / other committee sign off date) |  |
| Review Date |  |

| Version History | | |
| --- | --- | --- |
| Version | Date | Description |
|  |  |  |
|  |  |  |
| About this document   1. This template follows the guidance document: Ashmore et al., **A framework for developing Generic Implant Safety Procedures (GISPs) for scanning medical implants and devices in MRI**. Further details on developing a GISP can be found there. 2. The procedure statement provides a simple overview of the practical implementation of the GISP (typically completed last). 3. The Evidence review contains all the evidence which backs up the risk assessment and procedure statement (typically completed first). 4. The risk assessment provides an overview of the risk associated with the GISP 5. Roll over each section title for information on how to complete the section | | |

1. Table of Contents

[Example GISP Template 1](#_Toc164695577)

[1 Procedure Statement / Workflow 3](#_Toc164695578)

[2 Evidence Review 4](#_Toc164695579)

[2.1 Clinical context 4](#_Toc164695580)

[2.2 Results 4](#_Toc164695581)

[2.2.1 Online MRI implant safety databases (Date queried: DD/MM/YY) 4](#_Toc164695582)

[2.2.2 Locally implanting Teams (Date queried: DD/MM/YY) 4](#_Toc164695583)

[2.2.3 Implant manufacturers (Date queried: DD/MM/YY) 4](#_Toc164695584)

[2.2.4 Review of the peer reviewed literature (Date queried: DD/MM/YY) 4](#_Toc164695585)

[2.2.5 Internet search (non peer reviewed literature) (Date queried: DD/MM/YY) 4](#_Toc164695586)

[2.2.6 Regulatory Medical Device Databases (Date queried: DD/MM/YY) 4](#_Toc164695587)

[2.2.7 Regulatory Professional and Standards bodies (Date queried: DD/MM/YY) 4](#_Toc164695588)

[2.2.8 Anecdotal evidence (Date queried: DD/MM/YY) 4](#_Toc164695589)

[2.2.9 Local MR safety databases and empirical evidence (Date queried: DD/MM/YY) 4](#_Toc164695590)

[2.3 Discussion (optional) 4](#_Toc164695591)

[2.4 References 4](#_Toc164695592)

[3 Risk Assessment 5](#_Toc164695593)

[3.1 Hazards 5](#_Toc164695594)

[3.2 Description of Risk 5](#_Toc164695595)

[3.3 Existing precautions 5](#_Toc164695596)

[3.4 Level of Risk 5](#_Toc164695597)

# [Procedure Statement / Workflow](https://academic.oup.com/bjr)

[Disclaimer](https://academic.oup.com/bjr)

Compiled here are Generic Implant Safety Procedures (GISP’s) for MRI. While steps have been taken to minimise the risk of adoption of these procedures, it should be noted that these are not completely without risk. Health boards, integrated care systems, trusts or private medical institutions should consider carefully whether they wish to adopt these procedures. They should do so via their own governance process and the procedures should be reviewed prior to use. Any institutions use of this policy shall be done so at their own risk. If you are a patient reading this, then we strongly advise you to contact your healthcare provider directly with any concerns prior to attending for your scan, as approaches may vary. It remains the responsibility of the individual registered radiographer to apply their MRI knowledge and professional judgment to the situation under consideration. If there is any doubt regarding the safety of the patient then additional advice should be sought from e.g. the MR Responsible person, MRSE or the lead Clinician for MR safety.

[Brief description:](https://academic.oup.com/bjr)

[What the procedure covers:](https://academic.oup.com/bjr)

[What the procedure does not cover, including notable exceptions:](https://academic.oup.com/bjr)

[Advice summary:](https://academic.oup.com/bjr)

# [Evidence Review](https://academic.oup.com/bjr)

## [Clinical context](https://academic.oup.com/bjr)

## [Results](https://academic.oup.com/bjr)

### [Online MRI implant safety databases (Date queried: DD/MM/YY)](http://www.gisp.com/)

### [Locally implanting Teams](http://www.gisp.com/) (Date queried: DD/MM/YY)

### [Implant manufacturers](http://www.gisp.com/) (Date queried: DD/MM/YY)

### [Review of the peer reviewed literature](http://www.gisp.com/) (Date queried: DD/MM/YY)

### [Internet search (non peer reviewed literature](http://www.gisp.com/" \o "Provides list of manufacturers of devices and information on new devices on the market. Provides potential reports on any adverse incidents. Can provides procedure information from other MRI centres (e.g. hospital websites))) (Date queried: DD/MM/YY)

### [Regulatory Medical Device Databases (Date queried: DD/MM/YY)](https://accessgudid.nlm.nih.gov/)

### [Regulatory Professional and Standards bodies](https://www.accessdata.fda.gov/scripts/cdrh/cfdocs/cfmaude/search.cfm) (Date queried: DD/MM/YY)

### [Anecdotal evidence](http://www.gisp.com/) (Date queried: DD/MM/YY)

### [Local MR safety databases and empirical evidence](http://www.gisp.com/) (Date queried: DD/MM/YY)

## Discussion (optional)

## References

# [Risk Assessment](https://academic.oup.com/bjr)

## [Hazards](https://academic.oup.com/bjr)

## [Description of Risk](https://academic.oup.com/bjr)

## [Existing precautions](https://academic.oup.com/bjr)

## [Level of Risk](https://academic.oup.com/bjr)

| Risk Description | Likelihood | Consequence | Risk |
| --- | --- | --- | --- |
|  |  |  |  |

**Risk Matrix (example provided by NHS Greater Glasgow and Clyde)**

| Likelihood |  | Impact/Consequences | | |  |
| --- | --- | --- | --- | --- | --- |
|  | **Negligible** | **Minor** | **Moderate** | **Major** | **Extreme** |
| **Almost Certain** | Medium | High | High | V High | V High |
| **Likely** | Medium | Medium | High | High | V High |
| **Possible** | Low | Medium | Medium | High | High |
| **Unlikely** | Low | Medium | Medium | Medium | High |
| **Rare** | Low | Low | Low | Medium | Medium |

**Medium (Yellow) High (Orange) or Very High (Red) risks are unacceptable. A GISP should not be created where the risk is High or V High.**
